# The Copper-Zinc Balance in Hypertension: A Cross-Sectional Study From Bangladesh

**DOI:** 10.7759/cureus.118010

**Published:** 2026-10-16

**Authors:** Fahmida Sharmin, Tanusri Chakrabarty, Shohana Akter, Rimpi Romana, Md. Ashikul Islam, Raisa Badhan, Afrida Wasima, Md. Matiur Rahman

**Affiliations:** 1 Biochemistry, Sir Salimullah Medical College, Dhaka, BGD; 2 Biochemistry, Shaheed M. Monsur Ali Medical College, Sirajganj, Sirajgang, BGD; 3 Biochemistry, Sher-e-Bangla Medical College, Barisal, BGD; 4 Biochemistry, Ahsania Mission Medical College, Dhaka, BGD; 5 Neurology, National Institute of Neurosciences & Hospital, Dhaka, BGD; 6 Microbiology, National Institute of Burn and Plastic Surgery, Dhaka, BGD; 7 Biochemistry, City Medical College, Dhaka, BGD; 8 Biochemistry, Bangladesh Medical University, Dhaka, BGD

**Keywords:** blood pressure, copper, copper-to-zinc ratio, hypertension, oxidative stress, trace elements, zinc

## Abstract

Background: Hypertension (HTN) is a major public health problem, and disturbances in trace-element homeostasis may contribute to its pathogenesis. Copper (Cu) and zinc (Zn) are involved in antioxidant defense and vascular function, while their relative balance may be reflected by the copper-to-zinc (Cu/Zn) ratio.

Objective: The aim of this study was to evaluate the association of serum Cu, Zn, and Cu/Zn ratio with HTN among adults attending Bangladesh Medical University (BMU), Dhaka.

Methods: This cross-sectional comparative study was conducted in the Department of Biochemistry and Molecular Biology, BMU, among 100 adults aged ≥40 years, comprising 46 hypertensive and 54 normotensive participants recruited from the Cardiology and Medicine outpatient departments. Serum Cu and Zn levels were measured using standard spectrophotometric methods, and the Cu/Zn ratio was calculated. Lifestyle factors, excess salt intake, dyslipidemia, and body mass index were also assessed. Independent-samples t-test, Chi-square test, and Spearman’s correlation analysis were used as appropriate. Binary logistic regression was performed to determine the independent association of serum Zn and Cu/Zn ratio with HTN after adjustment for dyslipidemia and excess salt intake. A p-value of <0.05 was considered statistically significant.

Results: The median age was 50 years in both groups. Excess salt intake was significantly more common among hypertensive participants (27; 58.70%) than normotensive participants (19; 35.19%), p=0.019, as was dyslipidemia (23; 50.00% vs. 10; 18.52%, p=0.003). Serum Cu levels did not differ significantly between hypertensive and normotensive participants (101.42±29.12 vs. 94.14±24.92 µg/dL, p=0.187). In contrast, serum Zn levels were significantly lower in the hypertensive group (63.46±19.01 vs. 73.75±24.54 µg/dL, p=0.015), while the Cu/Zn ratio was significantly higher (1.53±0.61 vs. 1.22±0.49, p=0.007). Serum Zn showed significant negative correlations with systolic blood pressure (SBP) (ρ = -0.412, p<0.001) and diastolic blood pressure (DBP) (ρ = -0.210, p=0.036), whereas the Cu/Zn ratio showed significant positive correlations with SBP (ρ=0.412, p<0.001) and DBP (ρ=0.278, p=0.005). Serum Cu showed no significant correlation with either SBP or DBP. After adjustment for dyslipidemia and excess salt intake, the fourth quartile of the Cu/Zn ratio was independently associated with HTN, with approximately 3.7-fold higher odds than the first quartile (adjusted odds ratio=3.723, p=0.043), whereas serum Zn showed no independent association.

Conclusion: Serum Cu was not significantly associated with HTN, whereas lower serum Zn and a higher Cu/Zn ratio were observed among hypertensive individuals. The Cu/Zn ratio showed significant correlations with both SBP and DBP and remained independently associated with HTN after adjustment for selected confounders. The Cu/Zn ratio may therefore have potential as an additional marker of trace-element imbalance in HTN; however, larger prospective studies are required to establish its clinical and causal significance.

## Introduction

Hypertension (HTN) is a major contributor to the global burden of non-communicable diseases and remains an important public health concern, particularly in low- and middle-income countries. The number of adults with HTN has approximately doubled between 1990 and 2019, with two-thirds of affected individuals living in low- and middle-income countries [1,2]. In South Asia, the prevalence of HTN among urban adults ranges from 13% to 48%, while in Bangladesh, approximately 20% of adults were reported to have HTN in 2015 [3,4]. HTN is often asymptomatic but substantially increases the risk of stroke, heart failure, renal disease, and other vascular complications, highlighting the importance of identifying potentially modifiable factors associated with its development [2,5].

Increasing evidence suggests that trace elements may contribute to the pathogenesis of HTN through their effects on vascular function and oxidative stress. Copper (Cu) is an essential micronutrient involved in enzymatic and metabolic processes, including antioxidant defense and maintenance of vascular connective tissue [6,7]. However, excess Cu may promote oxidative stress through the generation of reactive oxygen species, potentially contributing to vascular injury and HTN [8]. The association between serum Cu and HTN remains inconsistent, with studies reporting both positive and negative associations, while a meta-analysis found no significant difference in serum Cu levels between hypertensive and normotensive individuals [9-12].

Zinc (Zn) is another essential trace element with important antioxidant and anti-inflammatory functions and may have a role in cardiovascular protection [13,14]. Zn deficiency may contribute to elevated blood pressure through increased renal sodium reabsorption and potentially increased salt intake [15,16]. However, findings regarding the relationship between serum Zn and HTN have also been inconsistent. Although several studies have reported lower serum Zn levels in hypertensive individuals, others have demonstrated positive or no associations [9,10,11,16].

Importantly, Cu and Zn interact physiologically, and their balance may be more informative than either element considered individually. Zn influences intestinal Cu absorption, while disruption of Cu-Zn homeostasis may contribute to oxidative stress, a mechanism implicated in HTN [17,18]. Despite this potential biological relevance, relatively few studies have evaluated serum Cu, Zn, and the Cu/Zn ratio simultaneously, and the existing evidence remains limited and inconsistent. Therefore, this study aimed to evaluate the association of serum Cu, Zn, and the copper-to-zinc (Cu/Zn) ratio with HTN.

## Materials and methods

This cross-sectional study was conducted in the Department of Biochemistry and Molecular Biology, BMU, over a period of one year from March 2021 to February 2022. The study population comprised adults aged ≥40 years of both sexes, including hypertensive and normotensive individuals attending the outpatient departments of Medicine and Cardiology at BMU. Participants diagnosed with HTN were enrolled in the hypertensive group, while normotensive individuals were enrolled in the normotensive group. Participants were selected using a non-random purposive sampling technique.

Sample size

The sample size was determined using the following formula:

$n = \frac{(Z_{\alpha} + Z_{\beta})^2(\sigma_1^2 + \sigma_2^2)}{(\mu_1 - \mu_2)^2}$

Based on the findings, the mean serum Cu level was considered to be 1.45 mg/dL with a standard deviation (SD) of 0.59 in the hypertensive group and 1.80 mg/dL with an SD of 0.24 in the normotensive group [10]. Considering a two-tailed significance level of 5% (Z_α_ = 1.96) and 90% statistical power (Z_β_ = 1.28), the calculated sample size was 35 participants in each group. However, a total of 100 participants were ultimately enrolled.

Selection criteria

Participants aged ≥40 years of either sex who were diagnosed as hypertensive or normotensive were eligible for inclusion in the study. Individuals with obesity, diabetes mellitus, chronic kidney disease, chronic liver disease, or malignancy were excluded. Participants with severe HTN with complications, smokers, chronic alcohol users, and pregnant women were also excluded. In addition, patients receiving oral contraceptive pills, angiotensin-converting enzyme inhibitors, diuretics, or Cu and Zn supplements were excluded from the study.

Data collection

Data were collected using a structured data collection sheet prepared in both English and Bengali. The questionnaire comprised three sections covering participants’ demographic and general information, HTN-related clinical information, and laboratory investigation results. Participants attending the outpatient departments of Medicine and Cardiology at BMU for treatment or follow-up were recruited using non-random purposive sampling according to the predefined eligibility criteria. After obtaining written informed consent, a detailed clinical history was recorded. Blood pressure was measured three times at 1-minute intervals after the participant had rested for 3-5 minutes in a quiet setting and had avoided smoking, caffeine, and exercise for at least 30 minutes. The mean of the second and third measurements was recorded as the blood pressure value. All participants underwent assessment of serum Cu and Zn levels.

Sample collection and laboratory analysis

Approximately 3 mL of venous blood was collected aseptically from the antecubital vein using a sterile disposable syringe and transferred into a plain red-top vacutainer. Following clot formation for approximately 30 minutes, the samples were centrifuged at 3000 rpm for 10 minutes, and the separated serum was used for biochemical analysis. Serum Cu was estimated by the 3,5-DiBr-PAESA method, while serum Zn was measured using the 5-Br-PAPS method. Both analyses were performed using an Indiko Plus autoanalyzer (Thermo Fisher Scientific, Finland) at the Department of Biochemistry and Molecular Biology, BMU, with appropriate quality control measures.

Statistical analysis

Data were analyzed using Statistical Package for the Social Sciences (SPSS) version 26.0 (IBM Corp., Armonk, NY). Continuous variables were assessed for normality using the Shapiro-Wilk test, and log transformation was performed when appropriate. Continuous data were expressed as mean ± SD or median with interquartile range, according to the distribution of the variables, while categorical variables were presented as frequencies and percentages. The chi-square test was used to compare categorical variables. For continuous variables, the independent-samples t-test or Mann-Whitney U test was applied as appropriate. Spearman’s rank correlation coefficient was used to assess the relationship of serum Cu, Zn, and the Cu/Zn ratio with blood pressure. Multiple logistic regression analysis was performed to determine the independent association of serum Zn and the Cu/Zn ratio with HTN. A p-value of ≤0.05 was considered statistically significant.

Ethical considerations

Ethical approval was obtained from the Institutional Review Board (IRB) of BMU (No. BSMMU/2021/6949, Date: 07/08/2021). Written informed consent was obtained from all participants after explaining the study objectives, procedures, potential risks, benefits, and implications. Participants were assured of confidentiality and their right to withdraw from the study at any time without consequences. Appropriate safety precautions were followed during blood collection, and each participant was assigned a unique identification number to maintain confidentiality and anonymity. No experimental drug or placebo was administered during the study.

## Results

This cross-sectional study was carried out in the Department of Biochemistry and Molecular Biology, BMU, to evaluate the association of serum Cu, Zn, and the Cu/Zn ratio with HTN. A total of 100 subjects (54 normotensive and 46 hypertensive) were enrolled in this study from the OPD of cardiology and the OPD of medicine, BMU.

The largest proportion of participants belonged to the 40-49 years age group (45), followed by 50-59 years (36) and 60-69 years (16). Only three participants were aged ≥70 years. The median age was 50 years in both the hypertensive and normotensive groups.

Among participants, females constituted the majority (59, 59%), while males represented the remaining proportion of the study population. In the hypertensive group, 25 (54.35%) participants were female and 21 (45.65%) were male, whereas in the normotensive group, 34 (62.96%) were female and 20 (37.04%) were male.

Table 1 shows that excess salt intake and dyslipidemia differed significantly between hypertensive and normotensive participants. Excess salt intake was more common among hypertensive participants than normotensive participants (27; 58.70% vs. 19; 35.19%, p=0.019), while dyslipidemia was also significantly higher in the hypertensive group (23; 50.00% vs. 10; 18.52%, p=0.003). In contrast, a sedentary lifestyle was reported by 23 (50.00%) of hypertensive and 37 (68.52%) of normotensive participants, but the difference was not statistically significant (p=0.060). Similarly, BMI distribution was comparable between the groups, with overweight being the predominant category in both hypertensive (38, 71.74%) and normotensive (33, 70.37%) participants (p=1.000).

**Table 1:** Comparison of lifestyle factors, salt intake, dyslipidemia, and BMI between hypertensive and normotensive groups (N=100) The chi-square test was used to determine the level of significance. ns, non-significant; s, significant.

Variables	Hypertensive (n=46) frequency (%)	Normotensive (n=54) frequency (%)	p-value
Sedentary lifestyle	-	-	χ²=3.549; p=0.060
Yes	23 (50.00)	37 (68.52)	-
No	23 (50.00)	17 (31.48)	-
Excess salt intake	-	-	χ²=5.527; p=0.019
Yes	27 (58.70)	19 (35.19)	-
No	19 (41.30)	35 (64.81)	-
Dyslipidemia	-	-	χ²=11.610; p=0.003
Yes	23 (50.00)	10 (18.52)	-
No	11 (23.91)	17 (31.48)	-
Unknown	12 (26.09)	27 (50.00)	-
BMI	-	-	χ²=0.023; p=1.000
Normal (18.5-22.9)	13 (28.26)	16 (29.63)	
Overweight (23–24.9)	33 (71.74)	38 (70.37)	

Figure 1 illustrates the distribution of serum Cu, serum Zn, and the Cu/Zn ratio among hypertensive and normotensive subjects. Serum Cu levels were comparable between the two groups (101.42±29.12 vs. 94.14±24.92 µg/dL, p=0.187). In contrast, serum Zn levels were significantly lower in hypertensive subjects than in normotensive subjects (63.46±19.01 vs. 73.75±24.54 µg/dL, p=0.015). The Cu/Zn ratio was significantly higher in hypertensive subjects (1.53±0.61 vs. 1.22±0.49, p=0.007). These findings indicate that HTN was associated with lower serum Zn levels and a higher Cu/Zn ratio, whereas serum Cu levels did not differ significantly between the groups.

**Figure 1:**
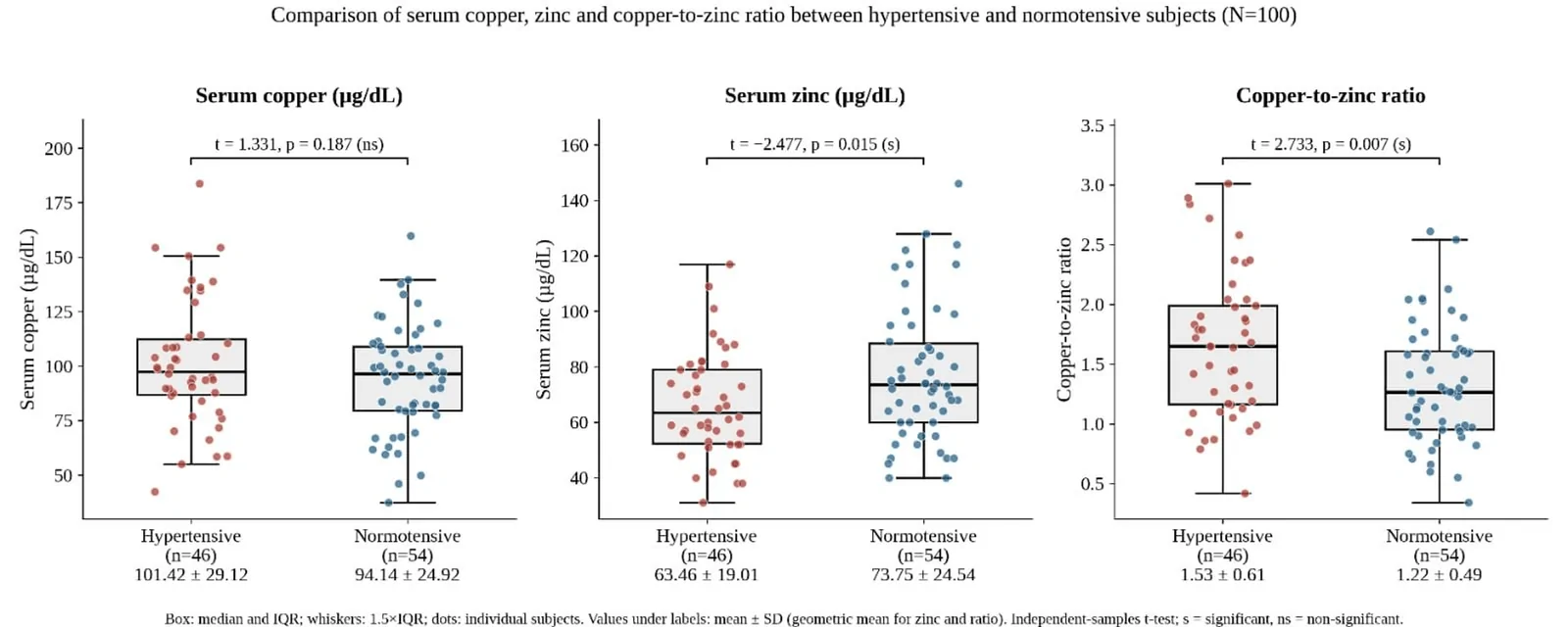
Comparison of serum copper, serum zinc, and copper-to-zinc ratio between hypertensive and normotensive subjects Box-and-whisker plots comparing serum copper (A), serum zinc (B), and copper-to-zinc ratio (C) between hypertensive (n=46) and normotensive (n=54) subjects. Boxes represent the interquartile range (IQR), horizontal lines within the boxes indicate medians, and whiskers extend to 1.5 × IQR. Individual dots represent participants. Values below the group labels are presented as mean ± standard deviation. Between-group comparisons were performed using independent-samples t-tests. Serum copper levels did not differ significantly between groups (t=1.331, p=0.187), whereas serum zinc levels were significantly lower (t = −2.477, p=0.015) and the copper-to-zinc ratio was significantly higher (t=2.733, p=0.007) in hypertensive subjects than in normotensive subjects. A p-value of <0.05 was considered statistically significant.

Each panel plots one subject per dot (N=100) with a linear trend line, for systolic blood pressure (SBP) (top row) and diastolic blood pressure (DBP) (bottom row). Red panels are significant correlations, and blue panels are non-significant ones. Serum Cu was not correlated with SBP (ρ=0.071, p=0.485) or DBP (ρ=0.081, p=0.422). Serum Zn was negatively correlated with SBP (ρ = −0.412, p<0.001) and DBP (ρ = −0.210, p=0.036). The Cu/Zn ratio was positively correlated with SBP (ρ=0.412, p<0.001) and DBP (ρ=0.278, p=0.005). Spearman's correlation coefficient was used. Points are slightly jittered vertically for visibility because blood pressure was recorded in discrete steps (Figure 2).

**Figure 2:**
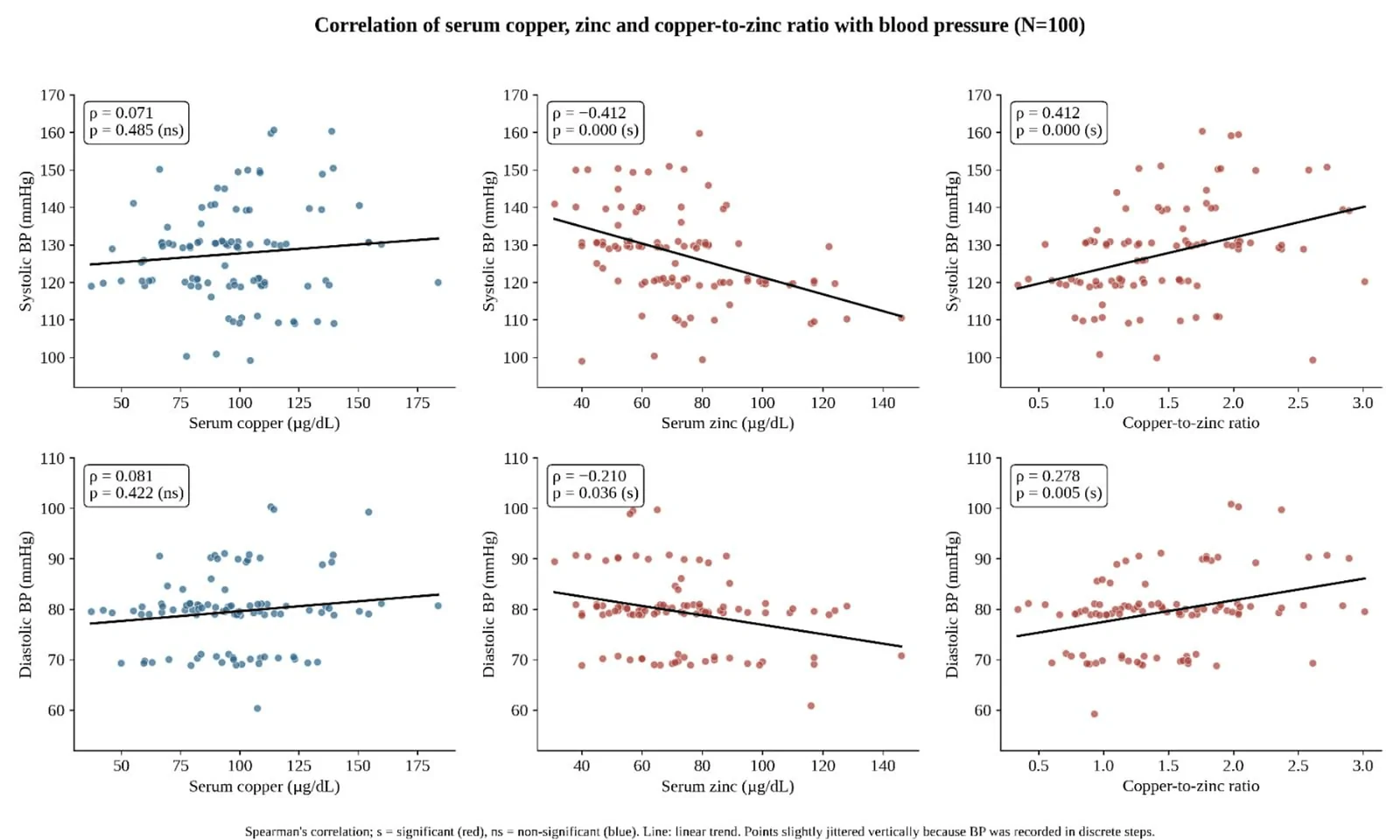
Correlation of serum copper, serum zinc, and copper-to-zinc ratio with systolic and diastolic blood pressure among study participants The upper row represents correlations with systolic blood pressure, and the lower row represents correlations with diastolic blood pressure. The columns show serum copper (left), serum zinc (middle), and copper-to-zinc ratio (right). Spearman’s rank correlation coefficients (ρ) and corresponding p-values are displayed within each panel. Black lines indicate linear trend lines. Red points indicate statistically significant correlations, whereas blue points indicate non-significant correlations. A p-value of <0.05 was considered statistically significant.

As shown in Table 2, serum Zn quartiles were not significantly associated with HTN after adjustment for dyslipidemia and excess salt intake. Compared with the fourth quartile, the first, second, and third quartiles had adjusted odds ratios of 2.453 (p=0.182), 3.237 (p=0.074), and 2.123 (p=0.249), respectively. Although the second quartile showed higher estimated odds, the association did not reach statistical significance.

**Table 2:** Adjusted odds ratios for hypertension according to serum zinc and serum copper-to-zinc ratio quartiles, adjusted for dyslipidemia and excess salt intake ^s^indicates statistical significance. Binary logistic regression analysis was performed with hypertension status (yes/no) as the dependent variable. The model was adjusted for dyslipidemia and excess salt intake. For serum zinc, the fourth quartile was used as the reference category, whereas for the serum copper-to-zinc ratio, the first quartile was used as the reference category. Reference categories were selected to facilitate comparison across the respective exposure distributions. β represents the regression coefficient, and the adjusted odds ratio represents the odds of hypertension relative to the corresponding reference category after adjustment for the specified covariates. Statistical significance was set at p<0.05.

	β	Adjusted odds ratio	p-value
Serum zinc	-	-	-
1^st^ quartile	0.89	2.453	0.182
2^nd^ quartile	1.17	3.237	0.074
3^rd^ quartile	0.75	2.123	0.249
4^th^ quartile	-	1.00 (ref)	-
Serum copper-to-zinc ratio	-	-	-
1^st^ quartile		1.00 (ref)	
2^nd^ quartile	0.69	2.013	0.248
3^rd^ quartile	0.95	2.608	0.141
4^th ^quartile	1.31	3.723	0.043^s^

In contrast, the serum Cu/Zn ratio demonstrated a statistically significant association with HTN in the fourth quartile. Compared with the first quartile, the fourth quartile had an adjusted odds ratio of 3.723 (p=0.043), indicating approximately 3.7-fold higher odds of HTN after adjustment for dyslipidemia and excess salt intake. The associations for the second and third quartiles were not statistically significant (p=0.248 and p=0.141, respectively). These findings suggest that a higher serum Cu/Zn ratio was associated with higher odds of HTN, whereas serum Zn alone showed no statistically significant association in the adjusted model. However, the findings should be interpreted cautiously because the sample size was relatively small and confidence intervals were not provided in the table.

## Discussion

HTN is a major global public health problem and an important cause of premature mortality. Its pathogenesis is complex and involves multiple interrelated mechanisms, among which the potential contribution of trace elements such as Cu and Zn remains incompletely understood. The present study therefore evaluated the association of serum Cu, Zn, and the Cu/Zn ratio with HTN among adults attending BMU, Dhaka.

In the present study, the hypertensive and normotensive groups were comparable in age and sex. A previous study also reported a similar age distribution between the groups [9]. In contrast, another study reported a greater likelihood of HTN with increasing age [19]. This discrepancy may partly be explained by differences in participant selection, as they included individuals aged ≥18 years [19], whereas the present study included only participants aged ≥40 years, thereby reducing the potential influence of younger participants in the normotensive group. Female participants predominated in both groups, with no significant difference in sex distribution, which is consistent with the findings of a previous study [20]. Conversely, another study, which included a wider age range of 8-80 years, reported male predominance among hypertensive participants [21]. The relatively high proportion of postmenopausal women in the present study may have contributed to the observed female predominance, as the risk of HTN increases after menopause [22].

Among the conventional risk factors, dyslipidemia and excessive salt intake were significantly more common among hypertensive participants. The association between dyslipidemia and HTN has been previously reported [23], while another study similarly demonstrated an association between higher salt intake and HTN [24]. In contrast, no significant difference was observed between the groups regarding physical activity or BMI. The absence of an association with physical activity may partly reflect lifestyle modification following a diagnosis of HTN. Similarly, the comparable BMI distribution between the groups may be related to the age composition of the study population, as overweight has been reported to be particularly prevalent among individuals aged 51-65 years [23].

Most participants had serum Cu and Zn levels within the conventional reference range; however, the Cu/Zn ratio was frequently elevated. This finding may reflect the relatively broad reference ranges of individual serum Cu and Zn concentrations and suggests that the ratio may provide additional information regarding the balance between these two trace elements.

The present study found no significant difference in serum Cu concentration between hypertensive and normotensive participants, and serum Cu was not significantly associated with blood pressure. This finding is consistent with a population-based study [19], which found no significant association between serum Cu and HTN. Similarly, a meta-analysis reported no significant overall difference in serum Cu levels between hypertensive and comparison groups [11]. However, other reported a nonlinear association, with both low (<80 µg/dL) and high (>130 µg/dL) serum Cu concentrations associated with increased risk of elevated blood pressure, while concentrations between 80 and 130 µg/dL were associated with lower risk [20]. These findings indicate that the relationship between Cu status and blood pressure may not be linear. Cu has an important physiological role in antioxidant defense and vascular function, including its involvement in superoxide dismutase activity and inhibition of angiotensin-converting enzyme [25]. Conversely, excessive Cu may exert pro-oxidant effects and promote oxidative stress [8]. Differences in dietary intake, ethnicity, geographic location, genetic background, study design, and analytical methods may therefore contribute to the inconsistent findings across studies.

In contrast to Cu, serum Zn levels were significantly lower among hypertensive participants and showed a negative correlation with blood pressure. Similar observations have been reported by some studies, while a meta-analysis demonstrated lower serum Zn concentrations in hypertensive individuals [9-11]. Nevertheless, after adjustment for potential confounders, serum Zn was not independently associated with HTN in the present study. Comparable findings have been reported in several studies [19-22]. These findings suggest that although lower Zn levels may be related to blood pressure, the observed association may be influenced by other interrelated factors rather than representing an independent relationship. Variations in ethnicity, study design, statistical approaches, and the ability to adequately control for confounding factors may explain the discrepancies among studies. Further large-scale prospective studies are warranted to clarify the temporal and causal relationship between Zn status and HTN.

A notable finding of the present study was the significantly higher Cu/Zn ratio among hypertensive participants. The ratio was positively correlated with blood pressure, and multivariable analysis demonstrated that participants in the fourth quartile of the Cu/Zn ratio had approximately 3.7-fold higher odds of HTN than those in the first quartile. A similar association was reported by a study of 2022 [26]. Compared with the individual assessment of Cu or Zn, the Cu/Zn ratio may better reflect the balance or homeostasis between these two trace elements [17]. An increased Cu/Zn ratio has also been associated with systemic oxidative stress [27], while oxidative stress is recognized as an important component of the pathophysiology of HTN [18]. Furthermore, higher Cu/Zn ratios have been associated with increased all-cause mortality in older populations, suggesting that this ratio may have broader clinical relevance [28].

Overall, the findings of this study suggest that serum Cu alone may not be significantly related to HTN, whereas lower serum Zn and, particularly, a higher Cu/Zn ratio may be associated with elevated blood pressure. The stronger relationship observed for the Cu/Zn ratio may reflect the importance of the balance between these trace elements rather than their individual concentrations. However, the cross-sectional design does not allow causal inference, and the relatively small sample size and single-center setting may limit the generalizability of the findings. Further large-scale prospective studies are needed to establish the clinical significance and potential mechanistic role of the Cu/Zn ratio in HTN.

## Conclusions

The present study demonstrated that the serum Cu/Zn ratio was significantly higher in individuals with HTN and remained independently associated with HTN after adjustment for potential confounders. Although serum Zn levels were significantly lower among hypertensive participants, this association was not independent after adjustment. Serum Cu levels showed no significant association with HTN. These findings suggest that the Cu/Zn ratio may provide additional information regarding the relationship between trace-element homeostasis and HTN.
